# Post-Transcriptional Regulatory Mechanism Based on CsrA and *rpoS* in Extremophile Sulfur Oxidizer *Acidithiobacillus caldus*

**DOI:** 10.3390/microorganisms14030724

**Published:** 2026-03-23

**Authors:** Yiwen Zhu, Panyan Chen, Hailin Yang, Yanjun Tong, Shoushuai Feng

**Affiliations:** 1 Key Laboratory of Industrial Biotechnology, Ministry of Education, School of Biotechnology, Jiangnan University, Wuxi 214122, China; 6230209032@stu.jiangnan.edu.cn (Y.Z.); 18306122558@139.com (P.C.); z19519939355@126.com (H.Y.); 2 State Key Laboratory of Food Science and Resources, School of Food Science and Technology, Jiangnan University, Wuxi 214122, China

**Keywords:** post-transcriptional regulation, *Acidithiobacillus caldus*, CsrA, RpoS, conjugative transfer

## Abstract

*Acidithiobacillus caldus* is perpetually exposed to multiple extreme environmental stresses. CsrA, functioning as a post-transcriptional regulator of physiological metabolism, acts as a differential modulator, facilitating more economical and efficient adaptation to extreme environments. The *csrA* expression recombinant strain was constructed in *A. caldus* MTH-04 by conjugative transfer technology pJD215. Physiological characterization revealed enhanced acid tolerance, significantly elongated flagella, elevated extracellular secretion, and altered biofilm composition. Notably, intracellular concentrations of free glutamate and aspartate increased to 24.18 mg/L and 16.07 mg/L, respectively. The secondary structure of CsrA protein was determined in vitro through circular dichroism spectroscopy and size-exclusion chromatography. Electrophoretic Mobility Shift Assay (EMSA) successfully demonstrated in vitro binding activity of CsrA to the *rpoS* leader mRNA. CsrA suppresses *rpoS* mRNA translation by competing with ribosomes for binding sites, thereby negatively regulating *rpoS* expression. Critical binding sites were further validated through site-directed mutagenesis. Through EMSA, RT-qPCR and the translation reporter system, it was also found that CsrA has a dual regulatory function for nearby flagella- and motility-related gene clusters (*flgC, 07035, motD, 15040*), which also implies the global regulatory role of CsrA. In summary, a potential overall post-transcriptional regulatory mechanism based on CsrA and *rpoS* by extremophile *A. caldus* was proposed. Finally, the efficiency of bioleaching application by *csrA* overexpression strain was improved by 20.81%.

## 1. Introduction

Bioleaching, a cost-effective and environmentally friendly technique harnessing microbial metabolism, has emerged as a promising alternative to traditional metallurgy for extracting metals from sulfide ores, especially those of low-grade quality [1]. Among the various microorganisms involved in biomining, *Acidithiobacillus caldus* (*A. caldus*) stands out due to its rapid growth rate, strong oxidation ability, and high tolerance to extreme environments. This bacterium can oxidize sulfide minerals, such as chalcopyrite, releasing metal ions into the solution. Additionally, the acidic metabolites produced by *A. caldus* lower the environmental pH, which not only promotes the degradation of sulfides but also significantly enhances the efficiency of metal extraction [2,3]. However, similar to other extreme microorganisms, *A. caldus* faces persistent environmental stresses that may compromise cellular integrity and suppress growth. To survive under such conditions, *A. caldus* has evolved sophisticated mechanisms to rapidly regulate its gene expression [4,5,6].

Post-transcriptional regulation plays a pivotal role in controlling bacterial gene expression, acting as a crucial checkpoint between transcription and translation to optimize the utilization of cellular resources [7,8]. By utilizing pre-existing transcripts, bacteria can quickly respond to environmental changes and fine-tune their gene expression patterns [9]. During stress responses, the selective degradation of non-essential mRNAs and the stabilization of stress-related mRNAs are essential for the efficient allocation of cellular energy and resources [10,11]. This regulatory process involves a complex network of molecular mechanisms, with RNA-binding proteins (RBPs) being key players [12]. RBPs, including Hfq, CsrA, and T3SS, are integral components of the co-transcriptional and post-transcriptional regulatory networks in bacteria [13,14,15,16]. These proteins, aside from ribosomes, can bind to mRNAs, often competing with ribosomes for binding sites, thereby influencing mRNA fate and gene expression. Most previous studies on post-transcriptional regulation in *A. caldus* have relied on heterologous expression systems, particularly *E. coli*. For example, Hu employed the CRISPR/Cas9 system to construct the recombinant strain Δ*hfq*/Ac*hfq*, successfully demonstrating the functional complementation of the *hfq*-deletion phenotype in E. coli and validating the function of the Hfq protein derived from *A. caldus* [17]. Similarly, the introduction of the molecular chaperone CbpA from *A. caldus* has been shown to enhance the acid tolerance of *E. coli* [18]. However, the in vivo mechanisms of these regulatory factors in *A. caldus* remain largely unexplored.

CsrA, a small yet highly conserved RNA-binding protein, exerts its regulatory functions by dimerizing and binding to specific GGA motifs in the 5′-untranslated regions (5′-UTRs) of target mRNAs [19]. This binding event can significantly alter the structure, translation efficiency, stability, and transcriptional elongation of the target mRNAs [20]. The carbon storage regulatory (Csr) network, conserved across a wide range of bacterial species, is involved in the regulation of multiple cellular processes, including carbon metabolism, iron homeostasis, motility, biofilm formation, stress response, c-di-GMP synthesis, quorum sensing, and virulence—associated factors [20,21,22,23,24]. In many bacteria, CsrA has been implicated in the regulation of environmental stress responses, often by modulating the expression of key regulatory factors such as the global stress sigma factors RpoS and AlgU. In *E. coli*, CsrA has been shown to regulate the translation efficiency and stability of *rpoS* mRNA through sequence-specific binding, thereby controlling the expression of a suite of stress-response genes [25]. In *Vibrio cholerae*, transcriptomic analysis and CsrA-RNA co-immunoprecipitation experiments have revealed that CsrA regulates approximately 22% of the entire transcriptome, including mRNAs encoding major sigma factors *rpoS* and *rpoE*, highlighting the broad-spectrum regulatory impact of CsrA [26]. Despite its importance, the exact function of the *csrA* gene and its post-transcriptional regulatory mechanisms in *A. caldus* remain elusive.

To address this knowledge gap, we first constructed *A. caldus csrA* overexpression strain to investigate the physiological effects of altered *csrA* levels on bacterial environmental adaptation. Using bioinformatics tools, we compared the structural and functional features of *A. caldus*-derived CsrA proteins with those of homologous proteins from other species. CsrA protein was successfully expressed and purified in *E. coli* BL21, and its dimer structure was determined. In vitro binding assay and reporter plasmid-based system were used to analyze the regulatory mechanism of CsrA on *rpoS* and explore its dual regulatory role in the flagella gene cluster. Finally, the *A. caldus csrA* overexpression strain was used to enhance the copper bioleaching efficiency of the strain, which provided theoretical guidance for practical industrial applications.

## 2. Materials and Methods

### 2.1. Strains and Plasmids

The strains and plasmids used in this study are listed in Table 1 and Table 2. The *Acidithiobacillus caldus* MTH-04 strain was cultured in Starkey medium at 40 °C and 180 rpm. Trace elements and metal salts were dissolved in sterile water and sterilized by filtration, while sulfur powder was sterilized by UV lamp irradiation. *Escherichia coli* strains were cultured in LB medium supplemented with kanamycin (50 µg/mL), ampicillin (100 µg/mL), and streptomycin (100 µg/mL) at 37 °C and 200 rpm. All genomic DNA and plasmids used in this study were extracted using kits from Nanjing Novizan Biotechnology Co., Ltd. (Vazyme, Nanjing, China).

### 2.2. Construction of the csrA Overexpression Strain

First, the Amp promoter and the *csrA* gene fragment were ligated by overlap extension PCR. Then, the recombinant plasmid pJD215-CsrA was constructed and transformed into *E. coli* SM10 competent cells. The *E. coli* SM10 (pJD215-CsrA) was activated overnight and then transferred to fresh medium. The culture was continued until the OD_600_ reached 0.6. An appropriate amount of the bacterial suspension was centrifuged, the supernatant was discarded, and the pellet was resuspended in a 1:1 inorganic salt solution. Meanwhile, *A. caldus* MTH-04 was cultured in Starkey-S^0^ liquid medium until the OD_600_ reached 1. An appropriate amount of the bacterial suspension was collected, and the sulfur powder was removed by low-speed centrifugation. After centrifugation to discard the supernatant, the pellet was resuspended in a 1:1 inorganic salt solution. The OD_600_ of the two bacterial suspensions was adjusted to be the same. Then, 1 mL of the donor bacteria and 2 mL of the recipient bacteria were mixed, centrifuged, and resuspended, and the mixture was spread on a solid conjugation medium covered with two 0.45 μm filter membranes and cultured upright at 37 °C for 5 days. Filters were rinsed with sterile inorganic salt solution and then serially diluted to 10^−1^, 10^−2^, and 10^−3^. The diluted suspensions were spread on Starkey-Na_2_S_2_O_3_ solid medium containing antibiotics and cultured at 37 °C for 7 days. Single colonies were picked from the solid plates and inoculated into 20 mL of Starkey-S^0^ medium. After 3–5 days of culture, 1 μL of the bacterial suspension was used for PCR verification, and the plasmid was extracted and sent for sequencing.

### 2.3. Determination of Bacterial Cell Concentration

In this experiment, the nucleic acid method was employed to determine the cell concentration of *A. caldus*. First, 1 mL of the culture medium was centrifuged at 3000 rpm for 30 s to remove sulfur powder. The supernatant was then mixed with 5% trichloroacetic acid at a 1:1 ratio. After incubating the mixture in a metal bath at 80 °C for 25 min, it was immediately cooled on ice. Using 5% trichloroacetic acid as a blank control, the absorbance of the samples was measured at OD_260_. Subsequently, the cell concentration was calculated according to the standard curve.

### 2.4. Observation of Bacterial Morphology

Transmission electron microscopy (TEM) (Hitachi, Ltd., Tokyo, Japan) was utilized to observe the morphology of the bacterial cells. Colonies cultured for 7 days were obtained from the surface of Starkey-Na_2_S_2_O_3_ solid plates. The colonies were fixed with 2.5% (*v*/*v*) glutaraldehyde for 0.5 h and then embedded in 1.25% (*v*/*v*) agar. The cell sections were subsequently soaked in a PBS solution containing 2.5% (*v*/*v*) glutaraldehyde for 0.5 h, followed by immersion in 1% (*v*/*v*) osmium tetroxide for 1 h. The sections were washed with ultrapure water and soaked in 1% (*v*/*v*) uranyl acetate solution for 1 h. Gradient dehydration was performed using ethanol. Finally, the stained agar sections were embedded in epoxy resin. The samples were observed using a transmission electron microscope.

### 2.5. Analysis of Biofilm Components Based on 3D-EEM

Biofilms were obtained using the sulfur chip method. The *A. caldus* culture in the logarithmic growth phase was adjusted to an OD_600_ of 1.0. The culture was statically incubated for 6 days. The sulfur chips were removed, gently washed twice with PBS buffer, air-dried, and treated with 1 mL of 33% glacial acetic acid to dissolve the biofilm. The 3D-EEM spectra of the samples were measured using an F-7000 fluorescence spectrophotometer (Hitachi, Ltd., Tokyo, Japan). The excitation wavelength (Ex) and emission wavelength (Em) were set in the range of 200 to 600 nm, with an increment of 5 nm and a scanning speed of 12,000 nm·min^−1^. 33% glacial acetic acid was used as a blank control. Rayleigh scattering was removed by clipping and baseline calibration in MATLAB R2022a (Version 9.121, MathWorks, Natick, MA, USA), and fluorescence regional integration (FRI) was employed for data analysis.

### 2.6. Determination of Intracellular Free Amino Acid Content

The wild-type and overexpression strains were cultured until the logarithmic growth phase. Bacterial cells with a wet weight of approximately 1.0 g were collected by centrifugation, washed 2–3 times with PBS buffer, and then fully resuspended in 1 mL of PBS buffer. The cell suspension was incubated in boiling water for 15 min. After centrifugation, the supernatant was collected. The supernatant was collected and mixed with 1 mL of 10% trichloroacetic acid solution. The mixture was thoroughly vortexed and allowed to stand for 10 min. The mixture was centrifuged again, and the resulting supernatant was collected. The resulting supernatant was collected and filtered through a 0.22 μm membrane filter. The intracellular free amino acid content was then determined using high-performance liquid chromatography (HPLC) (Agilent, Santa Clara, CA, USA).

### 2.7. Bioinformatics Analysis of the csrA Gene

The conserved regions and putative functions of the *csrA* gene were analyzed and identified using the NCBI online database (www.ncbi.nlm.nih.gov). For selected CsrA homologous proteins, Jalview software (www.jalview.org) was used for multiple sequence alignment, and Mega11 software (www.megasoftware.net) was employed to construct a phylogenetic tree. The ExPASy online database (www.expasy.org) was utilized to calculate physicochemical parameters and analyze the properties of CsrA. The SOPMA online website (NPS@: SOPMA secondary structure prediction (ibcp.fr)) was used to predict the secondary structure of the protein. Circular dichroism spectral data of the CsrA protein were analyzed using the Dichroweb online platform (https://bio.tools/dichroweb, accessed on 25 February 2026). The AlpaFold2 online tool (https://alphafold.com/, accessed on 25 February 2026) was used to simulate the spatial structure of the CsrA protein, and PyMoL 2.5 software (https://pymol.org/, accessed on 25 February 2026) was adopted to observe and interpret the tertiary structure of the protein.

### 2.8. Expression and Purification of CsrA Protein

The His-tagged CsrA (CsrA-H6) was purified according to the method described above [19].

### 2.9. Analysis of the Secondary Structure of CsrA Protein

The secondary structure of the CsrA protein was analyzed using circular dichroism (CD) spectroscopy. The desalting buffer was used as a blank control. The CD spectra were recorded from 190 to 250 nm at a scanning speed of 100 nm/min. The mean residue molar ellipticity ([θ]) was calculated using the following formula:

[θ] = mdeg × 1000/(l × c)/molecular weight of protein residues,

where l represents the optical path (mm), c represents the protein concentration (mM), and the unit of θ is deg·cm^2^·dmol^−1^.

To determine the spatial aggregation state of the CsrA protein, a Superdex-75 10/300 GL gel filtration chromatography column (Cytiva, Marlborough, MA, USA) was used with BSA (67.0 kDa), chymotrypsin (22.5 kDa), and cytochrome (12.4 kDa) as standard proteins. The mobile phase was desalting buffer (Tris-HCl), and the flow rate was 0.5 mL/min.

### 2.10. Electrophoretic Mobility Shift Assay (EMSA)

Primers were designed with the T7 promoter sequence (TAATACGACTCACTATAGGG) added to the 5′ end of the forward primer. The target gene fragment was amplified from the genome of *A. caldus* MTH-04 by PCR. After purification, it was used as a template to synthesize RNA in vitro using the T7 High-Yield RNA Transcription Kit (Vazyme, Nanjing, China). Prior to use, the RNA was heated at 65 °C for 5 min and then cooled to 4 °C. The RNA, CsrA protein solutions at varying concentrations, and binding buffer (10 mM Tris-HCl, 10 mM MgCl_2_, 100 mM KCl, 7.5% glycerol, 10 mM DTT, pH 7.5) were mixed and incubated at room temperature for 30 min to form the CsrA-RNA complex. The complex was then mixed with loading buffer. The mixture was loaded onto a 6% non-denaturing polyacrylamide gel with 0.5 × TBE solution as the buffer, and electrophoresis was carried out on ice for 2 h. After electrophoresis, the gel was stained with nucleic acid dye for 30 min and scanned using a gel imaging system.

### 2.11. Construction of Fluorescent Reporter Plasmids and Fluorescence Measurement

In this study, the pUC19 plasmid was used to construct translational reporter plasmids. Plasmid pAR1 contains a *Prpos-rpoS’-egfp* translational fusion (corresponding to nucleotides −402 to +18 relative to the rpoS start codon). Plasmid pAR2 was generated from pAR1 by deleting the nucleotide sequence from −341 to −1 using site—directed mutagenesis. Plasmid pAR3 contains the nucleotide sequence from −341 to −1 of the *rpoS* start codon. Plasmid pAR4 contains a PlacZ—*rpoS’*-*egfp* leader region fusion (including nucleotides +1 to +18 of the *rpoS* start codon). The pRSFDuet-1/pRSFDuet-*csrA* plasmids and the pAR series plasmids were co-transfected into *E. coli* BL21(DE3).

The pRSFDuet-1/pRSFDuet-*csrA* plasmids and PAR series plasmids were respectively transfected into *E. coli* BL21(DE3). When the strains containing the dual plasmids were cultured in LB liquid medium until the OD_600_ reached approximately 0.6, IPTG was added for induction for 16 h. The bacterial suspension to be tested was washed twice with PBS, and then the fluorescence was measured using a Synergy H4 microplate reader (BioTek, Winooski, VT, USA) at an excitation wavelength of 485 nm, an emission wavelength of 528 nm, a measurement height of 9.5 mm, and a gain value of 100. Fluorescence intensity was normalized to cell density using the formula: Fluorescence intensity = Fluorescence (AU)/OD_600_.

### 2.12. Application in Chalcocite Bioleaching Experiments

The strains were inoculated into Starkey liquid medium supplemented with 2 g/L FeSO_4_ and 10 g/L chalcocite powder, and incubated at 37 °C and 180 rpm for 30 days. Physicochemical parameters (pH, planktonic/attached biomass) were measured every 2–3 days. Bacterial concentrations were determined using the nucleic acid method. Samples were collected and centrifuged at low speed for 20 s to separate the supernatant and precipitate. The supernatant was further centrifuged at 12,000 rpm for 1 min to harvest the cells, which were then washed with PBS and finally treated with 1 mL of 5% trichloroacetic acid. The precipitate was washed with PBS, vortexed with 0.5 mL PBS to detach the bacteria attached to the sulfur powder, and centrifuged again at low speed for 20 s. The resulting supernatant was collected and treated with 0.5 mL of 5% trichloroacetic acid. OD_260_ was measured using a UV spectrophotometer (Yuanxi Instruments, Shanghai, China), and a standard curve was generated using the cell counting chamber method.

### 2.13. Determination of Copper in Bioleaching Process

The concentration of copper after bioleaching was determined by atomic absorption spectrophotometry, and the bioleaching system without biomass was used as the blank control group. In total, 1 mL of the test solution was centrifuged at 12,000 rpm for 1 min, and 100 μL of the supernatant was taken, 20 μL of concentrated nitric acid was added, 100 μL was removed and 10 mL was supplemented with ultrapure water. After microwave digestion, the samples were determined by a Varian AA-240 instrument (Varian, Palo Alto, CA, USA), and the standard curve was drawn using a standard copper solution.

### 2.14. Statistical Analysis

In this study, all experiments were performed in triplicate to ensure the reliability of the data. Statistical analysis was conducted using SPSS 25.0 software. One-way ANOVA and Student’s *t*-test were used to determine the significance of differences between groups, with *p* < 0.05 considered statistically significant.

## 3. Results

### 3.1. Construction of the csrA Overexpression Strains by Conjugative Transfer Technology and Analysis of Physiological Traits

#### 3.1.1. Establishment of the *csrA* Overexpression Strains and Their Cell Growth Patterns

**Figure 1:**
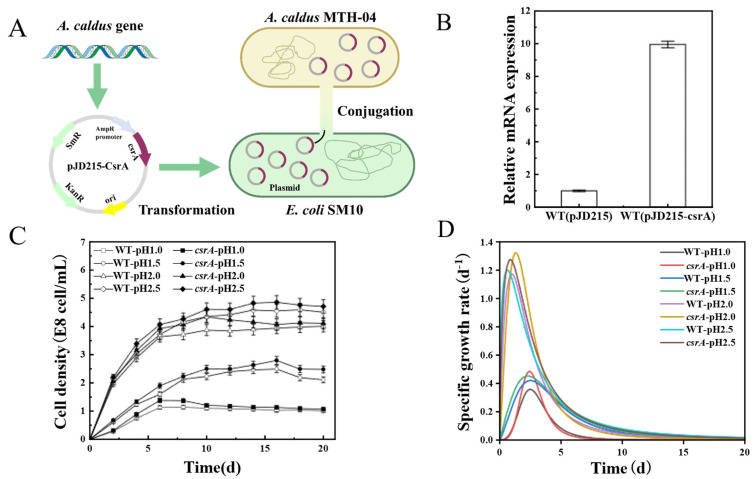
Construction verification and growth characteristic analysis of WT (pJD215-*csrA*) strain. (**A**) Schematic diagram of the construction of the overexpression plasmid pJD215-*csrA*; (**B**) detection of the expression level of the *csrA* gene by qRT-PCR; (**C**) growth curves of the overexpression strain and the control strain under different pH conditions; (**D**) specific growth rate curves. The error bars represented means ± standard deviations.

*A. caldus* has evolved to thrive in extremely acidic environments over a long period. The carbon storage regulator protein (CsrA) plays a critical role in modulating the acid stress response through multiple mechanisms, including transcriptional elongation, translation initiation, and the maintenance of RNA stability. In *A. caldus* MTH-04, a conserved region in the gene F0726_RS07045 was identified as belonging to the CsrA protein family, and the gene and its encoded protein were designated as *csrA* and CsrA, respectively. Due to difficulties in constructing the *csrA* gene-knockout strain, this research overexpressed the *csrA* gene. Initially, the overexpression plasmid pJD215-*csrA* was constructed following the schematic in Figure 1A. The recombinant plasmid was then introduced into the wild-type strain via conjugation transfer. After successful verification by sequencing, the resulting strain was designated as WT (pJD215-*csrA*). Fluorescence-based quantitative PCR analysis revealed that the expression level of the *csrA* gene in the overexpression strain was upregulated by a factor of 9 compared to the wild-type strain, thereby validating the successful construction of the overexpression strain.

The optimal growth pH for *A. caldus* MTH-04 was determined to be 2.5. In this study, acid-stress cultures were established at pH 1.0, 1.5, and 2.0. The cell growth patterns and specific growth rates were analyzed and are presented in Figure 1C,D. The results indicated that the wild-type strain exhibited the highest growth rate at pH 2.0, followed by pH 1.5, while the growth was severely inhibited at pH 1.0, with the maximum cell concentration reaching only 1.38 × 10^8^ cells·mL^−1^. Notably, under acidic conditions, both the growth yield and growth rate of *csrA* overexpression strains were significantly higher than those of the wild-type strain. After the fourth day under optimal conditions, differences in growth between the overexpression and control strains emerged. The maximum cell concentrations reached 4.85 × 10^8^ cell·mL^−1^ and 4.59 × 10^8^ cell·mL^−1^, with maximum specific growth rates of 1.20 d^−1^ and 1.27 d^−1^, respectively. Under the cultivation conditions of constant pH 2.0, 1.5, and 1.0, compared with WT, the maximum cell concentration of the *csrA* overexpression strains increased by 0.34 × 10^8^ cell·mL^−1^, 0.31 × 10^8^ cell·mL^−1^, and 0.23 × 10^8^ cell·mL^−1^, respectively. The maximum specific growth rate of the cells increased to 1.32 d^−1^, 0.45 d^−1^, and 0.48 d^−1,^ respectively. Over time, the cells entered the stationary phase. Due to the decrease in nutrients, the increase in stress, and the halt of growth, the cell concentration shows a downward trend.

#### 3.1.2. Cell Morphology Variations in the *csrA* Overexpression Strains

**Figure 2:**
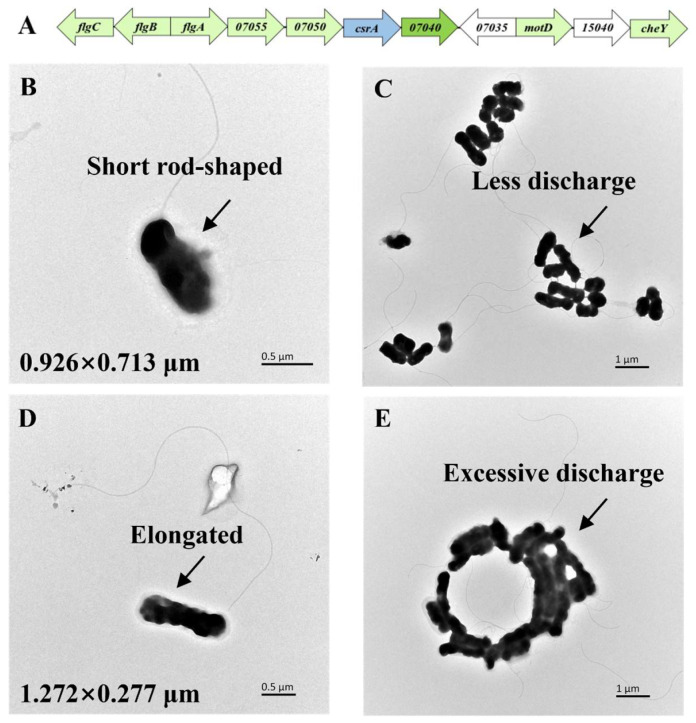
(**A**) Distribution of *csrA* gene and neighboring genes on the *A. caldus* genome; morphological observation of WT (**B**,**C**) and *csrA* overexpression strains; (**D**,**E**) under TEM.

In the genome of *A. caldus* MTH-04, the *csrA* gene is located within the flagellar coding gene cluster, and the periphery contains more than 40 genes related to flagella and chemotaxis, as shown in Figure 2A. Therefore, we hypothesized that CsrA protein might affect the motility and flagella structural composition of *A. caldus*. The flagella and cell morphology were observed by transmission electron microscopy (TEM).

As shown in Figure 2B, the flagellar structure of *A. caldus* MTH-04 is similar to that of most bacteria, consisting of a basal body, a flagellar hook, and a flagellar filament. A long, slender, and curved flagellum extends from one end of the cell [27]. Transmission electron microscopy (TEM) at various magnifications (Figure 2B–E) revealed three key differences between the *csrA* overexpression strains and the wild-type strain, which can be summarized as follows. The cell morphology shifted from rod-shaped to elongated form, and flagella became longer, while more extracellular substance was secreted. Specifically, the length of the *csrA* overexpression strains was 36.37% greater than that of the wild-type, while their width decreased.

#### 3.1.3. Effects of CsrA Overexpression on Biofilm Formation in *Acidithiobacillus caldus*

**Figure 3:**
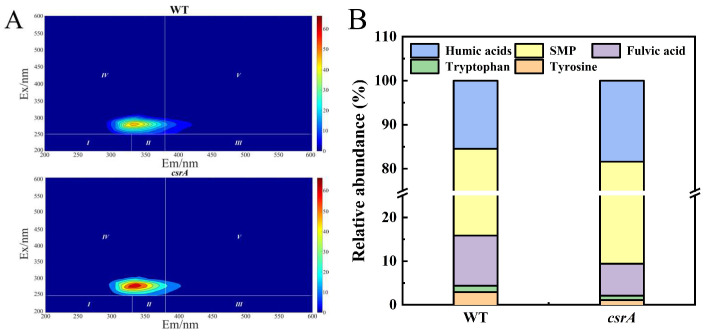
Overexpression of *csrA* gene affects *A. caldus* affects the composition of the biofilm. (**A**) 3D-EEM imaging of biofilms; (**B**) fluorescence integration values in different regions.

Biofilms are complex structures formed when microorganisms grow and adhere to solid surfaces, along with their extracellular secretions. For bioleaching microorganisms, biofilm formation is a critical adaptive response to extreme environmental conditions. To investigate the impact of *csrA* gene overexpression on biofilm formation in *Acidithiobacillus caldus*, this study utilized three-dimensional fluorescence spectroscopy to analyze biofilm samples grown on sulfur flakes. After removing background interference, including Raman and Rayleigh scattering using MATLAB, the spectral data shown in Figure 3A were obtained. Based on excitation and emission wavelengths, the biofilm components were categorized into five regions: Region I (tyrosine, an aromatic protein-like substance), Region II (tryptophan, an aromatic protein-like substance), Region III (fulvic acid-like substances), Region IV (soluble microbial by-products, SMP), and Region V (humic acid-like substances). The fluorescence regional integration method was applied to quantify the relative proportions of these components, as illustrated in Figure 3B. In the wild-type (WT) strain, the proportions of Regions I–V were 2.90%, 1.50%, 11.52%, 68.66%, and 15.50%, respectively. In contrast, in the *csrA* overexpression strains, the proportions were 1.10%, 1.0%, 7.30%, 72.19%, and 18.40%, respectively. Compared to WT, the *csrA* overexpression strain exhibited minimal changes in protein-like substances (Regions I and II), a reduction in fulvic acid-like substances (Region III), and an increase in soluble microbial by-products (Region IV) and humic acid-like substances (Region V).

#### 3.1.4. Differences in Intracellular Free Amino Acid Levels Due to CsrA Overexpression

In *Vibrio alginolyticus*, CsrA not only activates swarming motility but also regulates carbon and nitrogen metabolism, particularly amino acid metabolism. This enables CsrA to play a critical role in sensing and responding to environmental changes [20]. Based on the potential regulatory functions of CsrA, we hypothesize that in *Acidithiobacillus caldus*, CsrA may regulate branched-chain amino acids, amino acids involved in nitrogen metabolism, and amino acids associated with signal transduction and cellular functions to facilitate adaptation to environmental changes.

**Figure 4:**
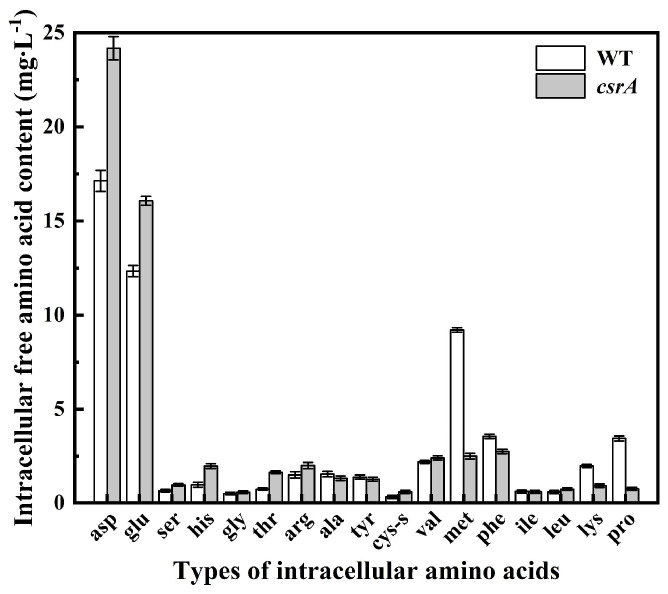
Changes in the content of free amino acids in the cells of the *csrA* overexpression strain. The error bars represented means ± SD.

The intracellular free amino acid levels in WT and *csrA* overexpression strains cells were measured, and the results are shown in Figure 4. Compared to the WT strain, the levels of aspartic acid and glutamic acid in the overexpression strain increased by 7.05 mg/L and 3.73 mg/L, respectively. This increase enhances cellular acid tolerance, thereby promoting cell survival. The levels of certain basic amino acids, such as histidine and arginine, also increased slightly, contributing to intracellular pH buffering. In contrast, the proline content decreased by 2.69 mg/L.

### 3.2. Identification of CsrA Protein Derived from Acidithiobacillus caldus

#### 3.2.1. Bioinformatics Analysis of the *csrA* Gene from *Acidithiobacillus caldus*

**Figure 5:**
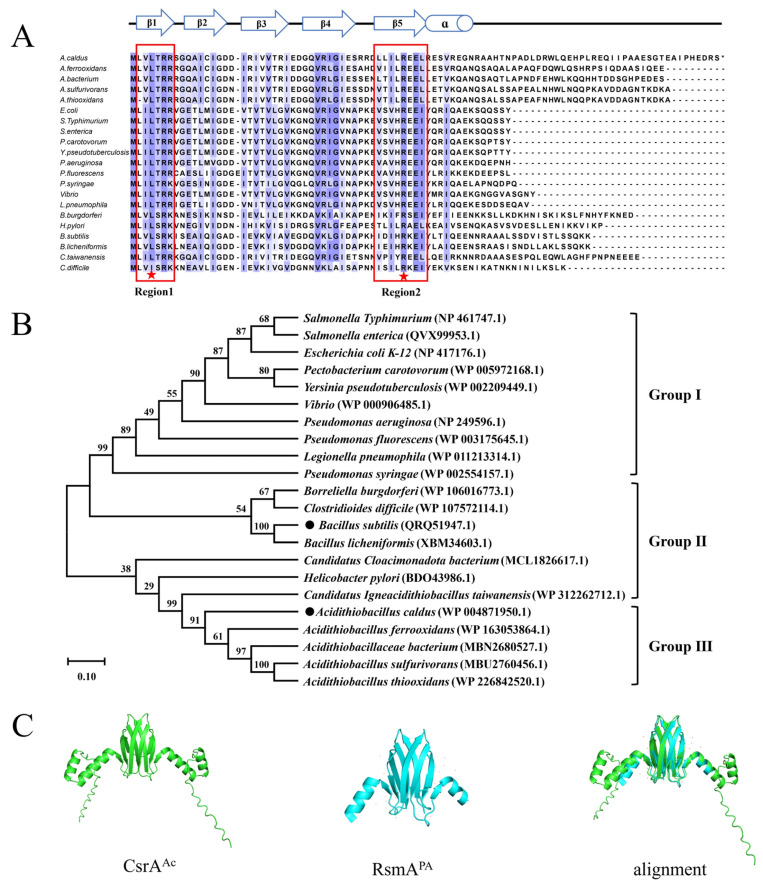
Bioinformatics analysis of CsrA. (**A**) Amino acid sequence alignment of the encoded protein of *A. caldus* MTH-04 and CsrA homologous proteins F0726_RS07045, the red stars indicate highly conserved regions; (**B**) evolutionary relationship of F0726_RS07045-encoded protein and homologous protein; (**C**) structural alignment between CsrA and RsmA proteins.

The *csrA* gene from *A. caldus* was analyzed through homologous protein sequence alignment with CsrA proteins from other representative strains. The results of the amino acid sequence alignment are depicted in Figure 5A. The CsrA protein from *A. caldus* comprises 95 amino acids and exhibits greater similarity to non-γ-proteobacteria.

Comprehensive alanine-scanning mutagenesis studies on the CsrA protein of *E. coli* have identified that the parallel regions of the β1 and β5 sheets are critical for RNA binding. Structural analyses of the CsrA-RNA complex have further confirmed that the amino acid residues essential for regulation directly interact with the bound RNA [19,28]. Importantly, conserved residues of the CsrA protein associated with RNA binding and in vivo regulation are highly conserved across different bacterial species. As illustrated in Figure 5A, the protein encoded by F0726_RS07045 also contains the conserved β1 and β5 regions, along with key amino acid residues, suggesting its potential for robust RNA-binding activity.

To further investigate the evolutionary relationships, typical model strains with high sequence similarity and extensive research background were selected. A phylogenetic tree was constructed using the Neighbor-Joining method in Mega-X, and the results are presented in Figure 5B. Based on the origin and autoregulation mechanisms of CsrA/RsmA, the strains were categorized into three groups. Group I includes typical γ-proteobacteria such as *E. coli*, *Vibrio*, and *Pseudomonas*, where the activation or inhibition of CsrA is mediated by non-coding sRNAs. Group II comprises strains like *Borreliella burgdorferi* and *Bacillus subtilis*, which possess a gene encoding the FliW protein near the *csrA* gene. The FliW protein inhibits CsrA activity through protein-protein antagonism. Group III consists of strains from the *Acidithiobacillus* genus. Phylogenetic analysis revealed that the protein encoded by F0726_RS07045 is highly conserved within the *Acidithiobacillus* genus and shows a relatively close relationship with *Bacillus subtilis* and *Pseudomonas aeruginosa* from Group II, sharing 48% sequence identity.

Using the phylogenetic tree results, the tertiary structure of the protein was predicted using the online tool AlphaFold2 v2.3.2. The well-characterized tertiary structure of RsmA from *Pseudomonas aeruginosa* in the CsrA/RsmA family was selected for comparison, and the PyMol 3.0.3 was employed to overlay the two protein structures. The results, shown in Figure 5C, demonstrate that the core region of the CsrA protein from *A. caldus* is highly conserved, with significant overlap in the β-sheets and additional helical structures at the C-terminus. Previous studies have indicated that the C-terminal α-helix of *E. coli* CsrA plays a key role in RNA recognition [29]. Therefore, it is plausible that the CsrA protein from *A. caldus* possesses strong RNA recognition and binding capabilities, although potential differences in regulatory mechanisms and functions may exist.

#### 3.2.2. Analysis of Secondary Structure and Oligomeric State of CsrA Protein

**Figure 6:**
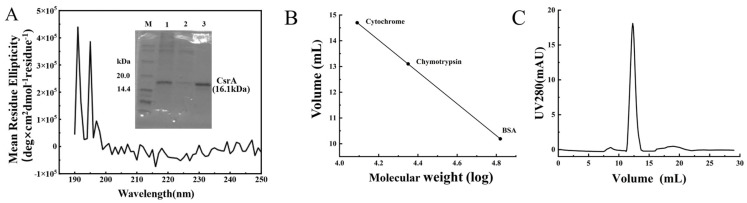
(**A**) Circular dichroism of *A. caldus* CsrA protein; (**B**) Standards for exclusion chromatography: cytochrome C (12.4 kDa); chymotrypsin (22.5 kDa); Bovine serum protein (67.0 kDa); (**C**) Exclusion chromatography of apo-CsrA^Ac^.

The recombinant plasmid pET28a-CsrA was constructed and subsequently transformed into the *E. coli* BL21 (DE3) strain to facilitate the heterologous expression and purification of the CsrA protein. In this study, the secondary structure of the target protein was analyzed using a high-precision circular dichroism (CD) spectrometer. According to bioinformatics predictions and previous studies, the secondary structure of CsrA and its homologs is predominantly composed of β-sheets [30]. Notably, the CsrA protein from *A. caldus* contains an additional, relatively long α-helix at its C-terminus. As shown in Figure 6A, the CD spectrum exhibits two distinct negative peaks at 222 nm and 208 nm, along with a positive peak at 191 nm, indicative of α-helical content. Furthermore, the negative peak at 218 nm and the positive peak at 196 nm provide strong evidence for the presence of β-sheets. To quantify the secondary structure composition, the CD data were analyzed using the Dichroweb online server. The results revealed that the CsrA protein consists of 16% α-helix, 39% β-sheet, 10.9% β-turn, and 34% random coil. These findings are consistent with the predictions from SOPMA, indicating that the recombinant protein is properly folded.

Proteins of the CsrA family are known to form dimers. In the simulated tertiary structure of *A. caldus* CsrA, the dimeric conformation closely resembles that of *Pseudomonas aeruginosa* RsmA. To experimentally validate the oligomeric state of CsrA, size-exclusion chromatography (SEC) was performed. A desalted protein solution was used as the sample, and the gel filtration column was calibrated using three standard protein samples to generate a molecular weight standard curve. As shown in Figure 6B,C, the theoretical molecular weight of CsrA is 16.1 kDa, while the SEC analysis yielded an apparent molecular weight of approximately 33.11 kDa. Therefore, *A. caldus* CsrA is confirmed to form a dimeric conformation.

### 3.3. Analysis of the Post-Transcriptional Regulation Mechanism of rpoS by CsrA Derived from Acidithiobacillus caldus

#### 3.3.1. CsrA Suppresses *rpoS* Expression Post-Transcriptionally

**Figure 7:**
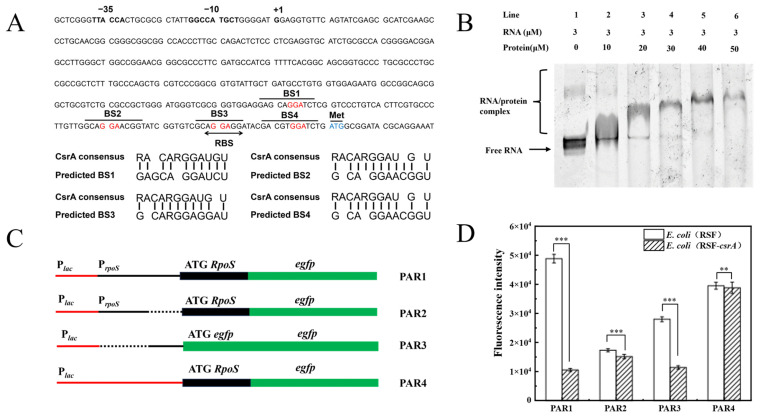
(**A**) *rpoS* promoter and pilot region with labeled −35 and −10 region promoter elements, transcription start site (+1), RBS sequence, and translation start codon (Met). The GGA motif of the potential CsrA binding site is indicated in red, and the sequence alignment of the CsrA binding site in the predicted *rpoS′* to the CsrA consensus sequence (R = A or G); (**B**) CsrA binds to *rpoS* lead RNA; (**C**) Schematic diagram of the fusion used in this analysis. The *rpoS* and *egfp* promoters, as well as the start codon, are indicated. The *lac* promoter is indicated by a thin red line, the *rpoS* leading region is represented by a thin black line, while the *rpoS* and *egfp* coding sequences are represented by a thick black line and a green line, respectively. The dotted line indicates that this part of the pilot region does not exist during the fusion process; (**D**) CsrA inhibits *rpoS* expression after transcription. The error bars represented means ± SD (D), the *p* value summary: **, *p *< 0.01; ***, *p *< 0.001.

The GGA motif is a well-characterized binding site for CsrA [31]. By predicting the position of the *rpoS* promoter, the *rpoS* leader region was identified as the nucleotide sequence spanning from the transcription start site to the start codon of the structural gene, designated as *rpoS′*. Multiple GGA motifs were identified within the *rpoS′* region, with potential CsrA binding sites highlighted in red in Figure 7A. The T7 promoter sequence (TAATACGACTCACTATAGGG) was incorporated into the design of conventional PCR primers. The *rpoS′* gene fragment was amplified via PCR, and the purified product served as a template for in vitro transcription using a T7 in vitro transcription kit, yielding *rpoSʹ* RNA.

To investigate the binding activity between CsrA and *rpoS′* RNA, a simplified EB staining method was employed in an Electrophoretic Mobility Shift Assay (EMSA). The concentration of *rpoS′* RNA was maintained at 3 μM, while the concentration of CsrA protein was incrementally increased from 0 μM to 50 μM. As shown in Figure 7B, a distinct retarded band appeared when the CsrA concentration reached 20 μM. With further increases in CsrA concentration, the retarded band progressively shifted upward, indicating enhanced binding activity.

To further elucidate the regulatory role of CsrA on *rpoS* expression, a series of translational reporter plasmids (PAR series) was constructed using the pUC19 plasmid as a vector. The schematic representation of these plasmids is illustrated in Figure 7C. The PAR1 translational fusion plasmid includes the sequence from −458 to +18 relative to the *rpoS* translation start site, along with the lac promoter. Subsequent deletions using the translational fusion method generated plasmids PAR2, PAR3, and PAR4. PAR2, a transcriptional fusion reporter plasmid, retains the promoter sequence from −458 to −342 within the *rpoS* leader region. PAR3 was derived by deleting the *rpoS* promoter region from PAR1, retaining only the leader-region sequence. PAR4 contains the sequence from +1 to +18 relative to the *rpoS* translation start site, designed to assess the impact of CsrA on the *rpoS* translation region.

The PAR1-PAR4 plasmids were transformed into *E. coli* BL21 strains harboring either the pRSFDuet-1-*csrA* plasmid (experimental group) or the pRSFDuet-1 plasmid (control group), establishing a two-plasmid system. Fluorescence detection results (Figure 7D) revealed that CsrA significantly inhibited the fluorescence expression of the translational fusion plasmids. Specifically, the expression level of PAR1 increased by 3.63-fold in the absence of CsrA, demonstrating that CsrA suppresses *rpoS* expression. In contrast, the expression levels of PAR2 and PAR4 remained largely unaffected by CsrA, while PAR3 exhibited a 1.45-fold increase in expression in the absence of CsrA.

#### 3.3.2. Regulatory Mechanism of CsrA on *rpoS*

**Figure 8:**
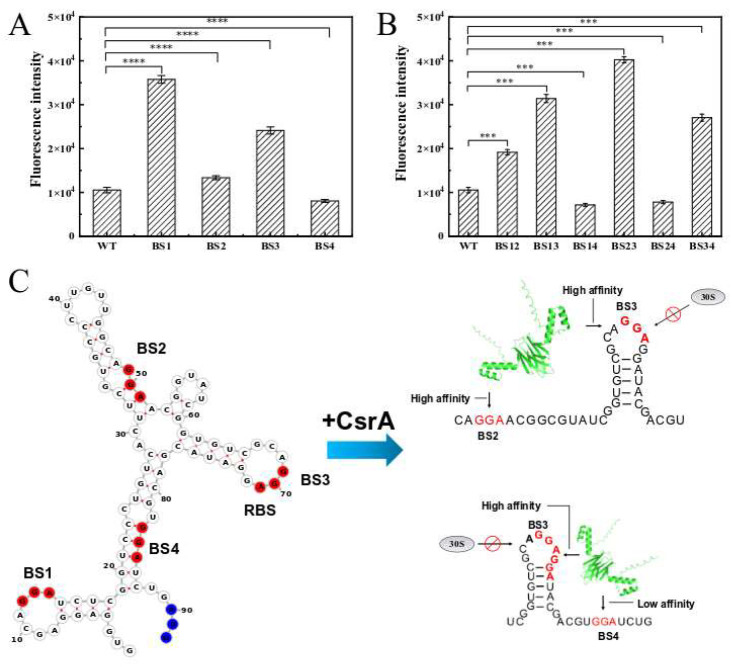
Translation fusion expression after GGA-to-GAA point mutation in BS sites and the predicted regulatory mechanism of CsrA. (**A**) Single-site mutation; (**B**) double-site mutation; (**C**) prediction of the secondary structure of *rpoS* leader region RNA and simulation of the CsrA action mechanism. On the left side is the schematic diagram of the secondary structure of *rpoS*′ mRNA, with the binding sites BS1, BS2, BS3, and BS4 labeled. The red-colored bases indicate the sites that may play an important role in the binding process. On the right side shows the situation after the CsrA protein binds to the RNA, and the high and low affinities of CsrA for the binding sites are labeled. The error bars represented means ± SD (**A**,**B**), the* p* value summary: ***, *p *< 0.001; ****, *p *< 0.0001.

Based on predictive analysis of the *rpoS* leader region mRNA, four potential binding sites for the CsrA protein (referred to as BS1–BS4) were identified. Experimental validation confirmed that CsrA post-transcriptionally inhibits *rpoS* expression. To further investigate the key binding sites of CsrA, this study utilized translation fusion plasmids for mutation analysis. Using site-directed mutagenesis PCR, the critical GGA motifs (highlighted in red) within BS1–BS4 were replaced with GAA. Subsequently, translation reporter plasmids containing these single-nucleotide substitutions were introduced into *E. coli* BL21 competent cells harboring the RSF-*csrA* plasmid. A dual-plasmid fluorescence reporter system was constructed, and the results are presented in Figure 8A. Mutations in BS1 and BS3 led to significant increases in fluorescence intensity by 239.13% and 128.71%, respectively, indicating a substantial reduction in the inhibitory effect mediated by CsrA. In contrast, mutations in BS2 and BS4 had no significant impact on fluorescence intensity.

Additionally, the study examined the effects of double-site mutations on *rpoS* expression. As shown in Figure 8B, mutations in BS12, BS13, BS23, and BS34 resulted in fluorescence intensity increases of 81.68%, 197.82%, 281.51%, and 156.4%, respectively. In comparison, mutations in BS14 and BS24 had relatively minor effects, further confirming that BS1 and BS3 are the primary CsrA binding sites. Notably, the BS1 site is located distal to the ribosome-binding site. It is hypothesized that mutations at BS1 may alter the secondary structure of the wild-type RNA, significantly impacting the inhibitory effect mediated by CsrA. Simultaneous mutations at BS1 and BS3 disrupt the RNA stem-loop structure, thereby attenuating the inhibitory effect.

#### 3.3.3. Interactions Between CsrA and the Leader Regions of Surrounding Gene

**Figure 9:**
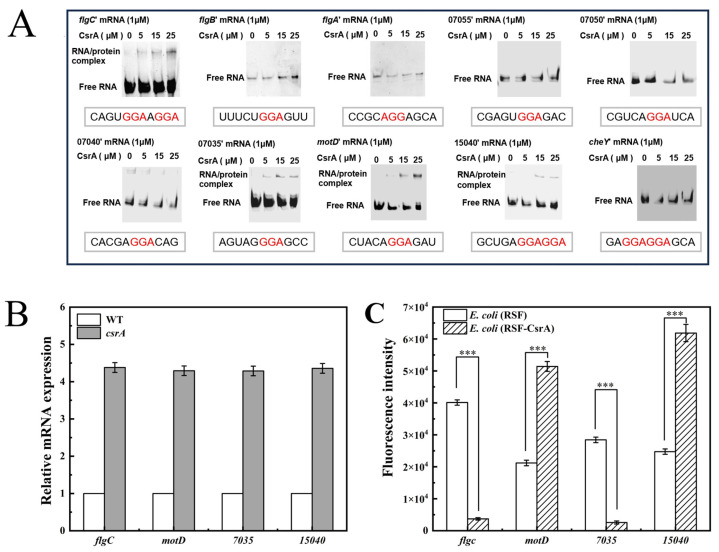
(**A**) Verification of CsrA’s binding activity to upstream and downstream gene lead regions; (**B**) RT-qPCR to verify the effect of CsrA on upstream and downstream gene transcription; (**C**) fluorescent reporter plasmid validates the effect of CsrA on upstream and downstream gene translation. The error bars represented means ± SD (**B**,**C**), the* p* value summary: ***, *p *< 0.001.

To further investigate the regulatory functions of *A. caldus* CsrA, five upstream and five downstream genes adjacent to *csrA* were selected as potential targets. The binding activity between the CsrA protein and RNA was validated in vitro using an Electrophoretic Mobility Shift Assay (EMSA). DNA fragments spanning the start codon of each gene (−180 to +20) were isolated and transcribed into mRNA in vitro. As shown in Figure 9A, the CsrA protein exhibited binding activity to the leader region RNAs of the *flgC*, *07035*, *motD*, and *15040* genes, as evidenced by distinct retardation bands in the gel electrophoresis.

Subsequently, the effects of CsrA on the expression of these target genes were assessed using Reverse Transcription Quantitative Polymerase Chain Reaction (RT-qPCR). The results, presented in Figure 9B, demonstrated that overexpression of CsrA led to varying degrees of upregulation in the expression of the four genes. This observed increase in gene expression may be attributed to two potential mechanisms: (1) CsrA may post-transcriptionally activate the translation of specific transcriptional regulatory factors, thereby indirectly influencing gene expression; or (2) CsrA may enhance mRNA stability, resulting in elevated mRNA levels. However, it is important to note that RT-qPCR results do not directly reflect changes at the translational level. In addition to modulating mRNA stability, CsrA can also inhibit protein synthesis by competing with ribosomes for binding sites [32].

To further elucidate the regulatory role of CsrA on target genes, a dual-plasmid fluorescence translation reporter system was constructed. As shown in Figure 9C, CsrA positively regulates the *motD* and *15040* genes. In contrast, CsrA exerts a negative regulatory effect on the *flgC* and *07035* genes. No significant binding activity was observed between the CsrA protein and the leader region RNAs of the remaining six genes in the gel electrophoresis analysis. This suggests that CsrA either does not regulate these genes or that its regulatory effect is too weak to be detected under the experimental conditions. Due to the unique cultivation requirements of *A. caldus*, RNA Immunoprecipitation (RIP) experiments could not be conducted at this time. As a result, numerous potential CsrA targets remain unidentified, and the in vivo regulatory functions of CsrA in *A. caldus* warrant further investigation.

### 3.4. Application of CsrA Overexpression Strain in Chalcocite Bioleaching

#### 3.4.1. Planktonic/Attached Biomass in Bioleaching Process

Our previous molecular and physiological characterization analysis showed that CsrA could play a dual role in improving the strain’s acid stress ability and regulating the expression of flagella and motility related gene clusters (*motD, 15040*). Next, the over-expressed strain was added into the bioleaching system to evaluate the colonization ability, stress resistance characteristics and final bioleaching efficiency of the overexpressed strain in the mineral matrix.

**Figure 10:**
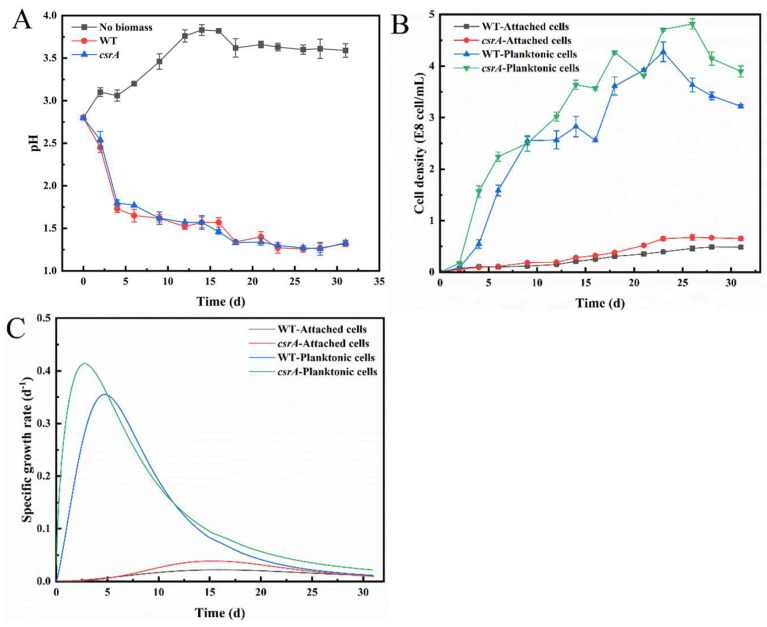
(**A**) Changes in pH of *A. caldus* WT and *csrA* overexpression strains in bioleaching systems; (**B**) Growth curves of planktonic/attached cells of *A. caldus* WT and *csrA* overexpression strains in different bioleaching systems; (**C**) The specific growth rates of planktonic/attached cells of *A. caldus* WT and *csrA* overexpression strains in different bioleaching systems.

As shown in Figure 10, the growth of the overexpressed strains was compared, and the phenomenon was generally consistent with the physiological characterization. The environmental pH of the chalcocite bioleaching system is about 2.8. With the increase in bioleaching time, the pH value decreases, and the pH value reaches about 1.3 in the later stage of bioleaching. The growth of WT was higher than that of WT under acidic bioleaching conditions. The overexpression strain increased rapidly in a short period of time. The highest planktonic cell concentration was 4.82 × 10^8^ cell·mL^−1^, and the specific growth rate *μ_max_* was 2.82 d^−1^. The concentration of attached cells was 0.68 × 10^8^ cell·mL^−1^, and the specific growth rate *μ_max_* was 15.17 d^−1^. Compared with the WT strain, the maximum planktonic cell concentration of the overexpression strain was increased by 0.54 × 10^8^ cell·mL^−1^, the maximum specific growth rate of the strain was increased to 1.83 d^−1^, the maximum attachment cell concentration was increased by 0.19 × 10^8^ cell·mL^−1^, and the maximum specific growth rate of the strain was increased to 0.5 d^−1^. With the increase in time, the bacteria entered a stable stage, and the concentration of bacteria showed a downward trend due to the decrease in nutrition, the increase in environmental pressure, and the growth arrest.

#### 3.4.2. Bioleaching Performance

**Figure 11:**
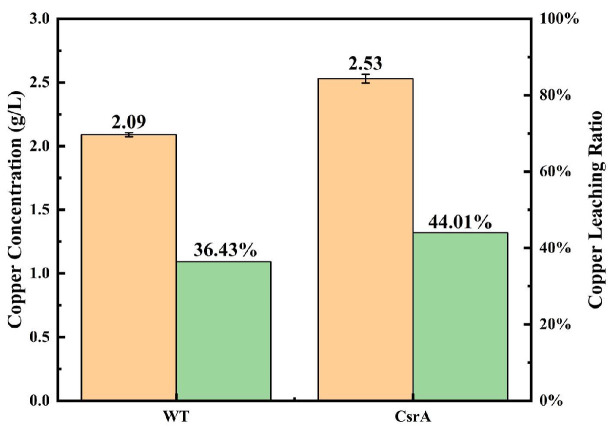
Bioleaching efficiency of WT and the *csrA* overexpression strains.

Upon reaching day 31 of the bioleaching process, the bacterial counts in each system stabilized and then began to decrease slightly, signifying the end of the bioleaching run. At this final point, the copper concentration in each system was determined to calculate bioleaching efficiency. As illustrated in Figure 11, the *csrA* overexpression strain exhibited a markedly higher copper bioleaching rate than WT. The final copper concentration and bioleaching efficiency for WT were 2.09 g/L and 36.43%, respectively. In comparison, the *csrA* overexpression strain achieved a copper concentration of 2.53 g/L and a bioleaching efficiency of 44.01%. This represents a significant improvement of 20.81% in bioleaching efficiency compared to WT.

## 4. Discussion

### 4.1. Analysis of Physiological Properties of csrA Overexpressed Strains

This study constructed a *csrA* expression recombinant strain of *Acidithiobacillus caldus* MTH-04, which exhibited increased acid tolerance. In *Vibrio cholerae*, the VarS/VarA-CsrA/B/C/D system regulates quorum sensing by controlling Qrr sRNA-related genes, and CsrA activity is modulated by carbon metabolites [33]. However, in *A. caldus*, no Csr-family non-coding regulatory sRNA has been found, and the quorum-sensing regulatory mechanism of CsrA remains unclear. Therefore, the enhanced growth observed under acidic conditions may be attributed to CsrA overexpression, which more effectively upregulates the expression of central carbon metabolism and transport genes, promoting metabolic adaptation and cell survival in extreme acid-stress environments. *A. caldus* was observed by TEM. The observed morphological differences between the *csrA* overexpression and the wild-type strains may be indirectly linked to CsrA’s regulation of endogenous glycogen accumulation, an effect most pronounced during the early stationary phase of cell growth. In *Pseudomonas syringae*, CsrA has been shown to promote cell division in response to environmental changes [34]. Additionally, Lovelock et al. identified MvaT as a target gene of RsmA through immunoprecipitation. In *Pseudomonas aeruginosa*, *mvaT* functions as a global regulatory gene, and its mutation leads to abnormal cell morphology [35]. This morphological change from an elliptical, plump form to a slender shape may provide *A. caldus*, a dominant ore-leaching bacterium, with advantages such as enhanced adhesion to mineral surfaces, improved nutrient utilization, and increased resilience to harsh environments. In addition, experimental results demonstrate that flagellar augmentation and extracellular secretions significantly increase in the *csrA* overexpression strains, indicating that CsrA enhances flagellar synthesis and motility in *A. caldus*. In *Escherichia coli*, CsrA overexpression promotes the synthesis of flagella and type I pili. Similarly, in *Helicobacter pylori*, CsrA has been shown to substitute for RpoF in positively regulating the expression of flagella-related genes (e.g., *flaA* and *flaB*) [36,37]. Furthermore, the increased extracellular secretions in the overexpression strain promote cell aggregation. CsrA regulates cell auto-aggregation through polysaccharide adhesins and indirectly modulates the transcription of operons involved in biofilm formation. Given the correlation between biofilm formation and cell aggregation [38], the enhanced aggregation of bioleaching microorganisms may increase the contact area between cells and ores, facilitating the accumulation of enzymes and metabolites. This, in turn, improves microbial resistance to environmental fluctuations and enhances the efficiency of metal leaching from ores.

Subsequently, we investigated the metabolic adaptations that support its increased acid resistance, including intracellular amino acid accumulation and structural characterization of CsrA. The observed decrease in fulvic acid content may slightly reduce biofilm stability and alter its permeability. However, the concomitant increase in humic acid content, a key and recalcitrant component of EPS, could partially compensate for these effects by enhancing biofilm stability. Humic acid exhibits strong complexation capabilities with metal ions such as Cu^2+^ and Cd^2+^. The resulting complexes can absorb onto the biofilm surface or become embedded within the EPS matrix. This process may form a physical barrier that impedes the diffusion of free Cu^2+^ toward the cell membrane, thereby enhancing the overall stress resistance of the biofilm and facilitating the attachment of bacteria to the mineral surface [39]. Humic substances are known to regulate microbial activity, particularly enzyme activity and gene expression, including genes encoding protease, redox enzymes, cellulase (*egl*), nitrate reductase (*narG*), and phosphatase (*phoA*). These substances also provide additional energy to microorganisms within the biofilm [40]. SMP, water-soluble metabolites such as organic acids and amino acids, are another key EPS constituent. These compounds can function as signaling molecules involved in quorum-sensing (QS) systems, thereby promoting biofilm formation on mineral surfaces and modulating community behavior. For example, a study on *Synechocystis* demonstrated that changes in SMP content can alter microbial community structure, such as reducing the abundance of *Sphingobacteriales* while increasing *Comamonadaceae* [41]. Therefore, the increased content of SMP and humic acid in the EPS enhances the formation of biofilms by the strain on the mineral surface. In *Acidithiobacillus ferrooxidans*, increasing Cu^2+^ concentrations have been shown to stimulate the production of EPS components [42]. It is speculated that the abundant copper ions in the bioleaching system can further enhance the EPS of the strain. The *csrA* overexpression strain exhibited greater mineral surface colonization, biofilm stress resistance, and environmental stress tolerance in the leaching system, resulting in increased strain activity and potentially improved bioleaching efficiency. Free amino acid levels were further analyzed. Glutamic acid undergoes a decarboxylase reaction to produce a positively charged product. During decarboxylation, protons are continuously consumed, which helps stabilize intracellular pH. Aspartic acid can be converted into alanine by consuming intracellular hydrogen ions, and ATP is subsequently generated through glycolysis and the tricarboxylic acid cycle, providing energy to maintain intracellular pH [43]. Proline accumulation is critical for osmotic stress tolerance. Proline accumulates within cells, increasing cytoplasmic solute concentration and intracellular osmotic pressure, thereby reducing water loss. This helps maintain cellular water balance, protects intracellular macromolecules, minimizes protein denaturation and nucleic acid damage under stress conditions, and preserves cellular functions [44]. Overall, CsrA overexpression leads to the accumulation of glutamate and aspartate, which enhances the survival of *A. caldus* under acidic stress conditions.

### 4.2. Identification of CsrA Protein of Acidithiobacillus caldus and Analysis of Its Molecular Regulatory Mechanism

In this study, sequence alignment, phylogenetic analysis and structural prediction revealed that the CsrA protein from *A. caldus* is highly conserved in amino acid sequence, key RNA binding site and core β-fold structure, but it belongs to an evolutionarily independent third group with a special helix structure at the C terminus. This suggests that its regulatory mechanism may be unique. Furthermore, structural analysis by circular dichroism spectroscopy and size-exclusion chromatography revealed that CsrA is in a dimeric conformation with a monomer composed mainly of β-folds with minor α-helices and random coils. These results confirm that *A. caldus* CsrA exists as a dimer in solution, which is consistent with the oligomeric state reported for CsrA from *Pseudomonas putida* [45].

Moreover, electrophoretic mobility shift assay (EMSA) confirmed that CsrA binds to the leader region of *rpoS* mRNA, inhibiting its translation by competing with ribosomes. These results suggest that the inhibitory effect of CsrA is independent of the promoter and transcription-start region, occurring post-transcriptionally. CsrA primarily interacts with the mRNA of the *rpoS* leader region to exert its regulatory function. Deletion of RpoS has previously been shown to up-regulate flagellar function and chemotaxin-related genes, and significantly enhance bacterial motility, indicating the inhibitory effect of RpoS on motility [46]. It was thus speculated that the negative regulation of *rpoS* by CsrA after transcription could enhance *A. caldus* motility to some extent. Further investigation of the binding mechanism revealed that the key binding motif was BS3 GGA by site-directed mutagenesis. In *E. coli*, the canonical inhibitory mechanism of CsrA involves binding to the GGA motif in the *glgC* leader region mRNA, competing with the 30S ribosome for the binding site to suppress *glgC* translation. The CsrA dimer initially binds to a high-affinity site within the single-stranded region of the RNA hairpin structure. Subsequently, its second RNA-binding surface interacts with a low-affinity site overlapping the Shine-Dalgarno (SD) sequence, thereby blocking ribosome binding [47]. Analysis of the *rpoS* leader region revealed that BS3 is a critical region for ribosome binding. Therefore, this study proposed a mechanistic model for the interaction between CsrA and *rpoS*, as illustrated in Figure 8C: in the presence of CsrA, the protein preferentially binds to the high-affinity BS3 site. To stabilize its structure, the second RNA-binding surface of CsrA may interact with the relatively high-affinity BS2 site or the low-affinity BS4 site. This multi-site binding regulatory mechanism enables CsrA to exert a more stable regulatory effect. For instance, in E. coli, small non-coding RNAs containing multiple high-affinity CsrA-binding sites (e.g., CsrB or CsrC) can sequester CsrA, thereby modulating its activity [48].

**Figure 12:**
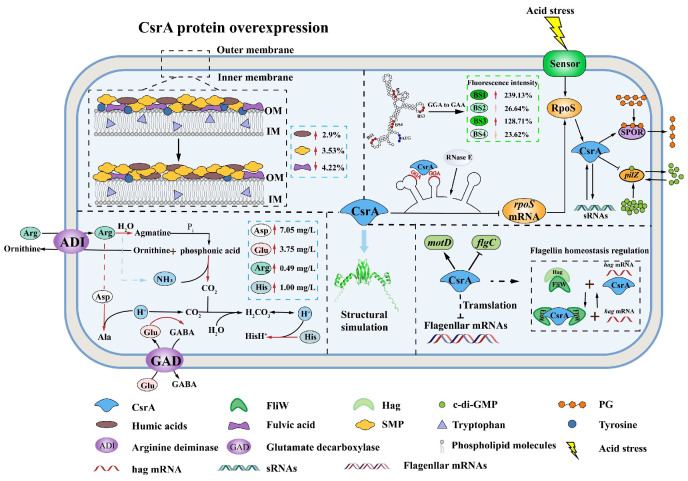
A potential post-transcriptional regulatory model of CsrA in *A. caldus*.

CsrA was found to exert dual functional effects, modulating the expression of flagella and motility-associated gene clusters (*flgC, 07035, motD, 15040*), underscoring its global regulatory role in *A. caldus*. Among them, the *motD* gene encodes the flagellar motor protein MotD, while the *15040* gene encodes a protein containing the SPOR domain. The bacterial SPOR domain binds to septal peptidoglycan (PG) at sites where cell wall amidases remove stem peptides, targeting the protein to the divisome and playing a critical role in cell division [49]. Initially identified as a repressor of gene expression during the stationary growth phase, CsrA is now speculated to be involved in regulating bacterial motility, primary metabolic pathways, and cell division in *A. caldus*. The *flgC* gene encodes the flagellar basal-body rod protein FlgC, and the *07035* gene encodes a protein containing the PilZ domain. In *Pseudomonas*, FlgC is implicated in the regulation of chemotaxis, motility, biofilm formation, and adhesion. Thus, it is hypothesized that CsrA differentially regulates the synthesis of bacterial flagellar structures [50]. Consequently, *A. caldus* CsrA may influence biofilm formation, primary metabolic pathways, and cell division. Based on the physiological characterization and post-transcriptional regulatory mechanisms explored in this study, a potential regulatory model of *A. caldus* CsrA is proposed and summarized in Figure 12.

### 4.3. Analysis of the Application of the csrA Overexpression Strain in the Bioleaching of Chalcocite

Based on the global regulatory mechanism of CsrA, this strain was applied to the bioleaching system of chalcocite. During the bioleaching process, a large number of strains closely adhered to the surface of chalcocite through the “contact” mechanism and continued to act, eventually driving the oxidation and dissolution of sulfide minerals, and achieving a substantial improvement of copper bioleaching efficiency [51]. This phenomenon aligns with the fundamental role of bacterial attachment in facilitating mineral dissolution. In the bioleaching process, *Acidithiobacillus ferrooxidans* tightly adheres to the mineral surface by secreting EPS. This attachment mechanism not only creates a microenvironment conducive to oxidation reactions but also promotes electron transfer and interfacial interactions between the bacteria and the mineral, thereby effectively driving the oxidative dissolution of sulfide minerals and significantly enhancing the bioleaching efficiency of target metals [52]. The biomass and growth rate of the *csrA* overexpression strain were higher than those of WT. This suggests that overexpression of *csrA* enhances bacterial environmental stress resistance by regulating genes involved in acid stress resistance, flagella formation and motility. Therefore, the overexpression strain showed stronger colonization ability and increased biomass accumulation, suggesting that this may enhance copper bioleaching efficiency through the intensification of contact mechanisms and metabolic activity.

Finally, the copper bioleaching efficiency of each strain was evaluated. The results showed that the bioleaching efficiency of the *csrA* overexpression strain was significantly increased by 20.81% compared to WT. In the chalcocite bioleaching system, the acid stress resistance mediated by CsrA effectively increased the biomass of the strain, enabling it to maintain normal physiological metabolism in a continuously decreasing pH environment, thereby avoiding cell lysis and growth arrest caused by environmental stress. This finding is consistent with previous studies, which demonstrated that the removal of attached cells weakens the “contact” bioleaching mechanism and inhibits sulfur/iron metabolic activity, consequently leading to a significant decrease in copper bioleaching efficiency [53]. In summary, this study provides an important theoretical basis and research direction for the modification of bioleaching industrial strains and the improvement of bioleaching efficiency.

## 5. Conclusions

This study successfully constructed a CsrA-overexpression strain of *Acidithiobacillus caldus* to systematically investigate its role in extreme environmental adaptation and application in chalcocite bioleaching. Physiological characterization revealed that CsrA overexpression significantly enhanced acid tolerance, induced flagellar elongation, increased extracellular secretions, and altered biofilm composition. Concurrent accumulation of free amino acids like glutamate and aspartate helped maintain intracellular pH homeostasis, boosting stress resistance. Mechanistic analysis demonstrated that CsrA exists as a dimer and post-transcriptionally represses *rpoS* expression by binding to a key GGA motif (BS3) in the *rpoS* mRNA leader, thereby competing with ribosomes. Furthermore, CsrA exerted dual regulatory effects on flagella and motility-related gene clusters (*flgC*, *motD*), underscoring its global regulatory role. Leveraging these mechanisms, the *csrA* overexpression strain exhibited improved growth and resilience in chalcocite bioleaching systems, ultimately enhancing copper bioleaching efficiency by 20.81%. This research elucidates a global post-transcriptional regulatory network in an extremophile and provides a strategic basis for strain improvement to enhance bioleaching efficiency.

## Figures and Tables

**Table 1:** Strains and plasmids used in this study.

Strains or Plasmids	Genotype or Description	Source
*A. caldus* MTH-04	Wild-type	This lab
*E. coli* DH5α	Cloning strain	This lab
*E. coli* BL21 (DE3)	Expression strain	This lab
*E. coli* SM10	Km^r^ thr leu hsd recA::RP4-2-Tc::Mu	This lab
pUC19	ori ColE1, Ap^r^, cloning vector	This lab
pAR1	pUC19 containing P*_rpoS_*-*rpoSʹ*-*ʹlacZ* (−402 to +18)	This study
pAR2	pUC19 containing P*_rpoS_*-*rpoSʹ*-*ʹlacZ* (−402 to −342, +1 to +18)	This study
pAR3	pUC19 containing P*_rpoS_*-*rpoS*-*ʹlacZ* (−341 to −1)	This study
pAR4	pUC19 containing P*_lacZ_*-*rpoSʹ*-*ʹlacZ* (+1 to +18)	This study
pRSFDeut-1	Kan^r^, ori RSF, T7 promoter, lacI^q^	This lab
pRSFDuet-1-CsrA	pRSFDuet-1 containing the *csrA* gene	This study
pET-28a (+)	Original plasmid utilized for protein expression	This lab
pET28a-CsrA	pET-28a (+) containing the *csrA* gene	This study
pJD215	Kan^r^, ESF1010 oriV, Expression vector	This lab
pJD215-CsrA	pJD215 containing the *csrA* gene	This study

**Table 2:** Primers used in this study.

Primer Name	Sequence (5′ to 3′) ^a^
pET28a-F	GGACAGGTCATGAAGCTTGCGGCCGCAC
pET28a-R	CGCGTGAGTACCAGCACAGCTTGTCGACGGAGCTCGAA
pET28a-7045-F	GCTCCGTCGACAAGCTGTGCTGGTACTCACGCGTC
pET28a-7045-R	GTGCGGCCGCAAGCTTCATGACCTGTCCTCGTGCG
pAR1-F	TTGGCGTAATCATGGTCATAGCTG
pAR1-R	TGCATGCCTGCAGGTCGA
pAR1-*rpoS*′-F	CATGATTACGCCAATGCTGGAGGTACTGCTGC
pAR1-*rpoS*′-R	GATCCACGTCGTATCCTCCTGC
Overlap-*egfp*-F	GGATACGACGTGGATCATGGGTAAGGGAGAAGAA
Overlap-*egfp*-R	GCAGGCATGCAGGCCGCAAATTAAAGCCTTCG
pAR2-F	GCTATTGGCCATGCTGATGGGTAAGGGAGAAGA
pAR2-R	TCTTCTCCCTTACCCATCAGCATGGCCAATAGCGCG
pAR3-F	GATTACGCCAAAGCTTGGGATGGAGGTGTTCAG
pAR3-R	ACTGAACACCTCCATCCCAAGCTTTGGCGTAATCATGGTCAT
pAR4-F	ATTACGCCAAAGCTTATGGCGGATACGCAGGAAATGGGTAAGGGAG AAGAACTTTTCACT
pAR4-R	AAGTTCTTCTCCCTTACCCATTTCCTGCGTATCCGCCATAAGCTTTGGCGTAATCATGGTCATAG
T7-*rpoS*′-F	TAATACGACTCACTATAGGGATGCTGGAGGTACTGCTGCAG
*rpoS*′-R	GATTTCCTGCGTATCCGCC
T7-*flgC*′-F	AATTAATACGACTCACTATAGGGGCCTTCGTCCAGTATCAGCG
*flgC*-R	CATTCGTACACCCCCCGAAG
T7-*flgB*′-F	AATTAATACGACTCACTATAGGGGCCAGGATTTTTTCTCGACGACG
*flgB*′-R	CATGGGCAGAACTCCAGAAAACT
T7-*flgA*′-F	AATTAATACGACTCACTATAGGGATGGGCTTATGTCGAGGTCAT
*flgA*′-R	CACCTTTCTGTAACCTCACGCA
T7-07055′-F	AATTAATACGACTCACTATAGGGGGCGCAAGCCATAGAAAACGG
07055′-R	CATGACGGATGTCTCCACTCG
T7-07050′-F	AATTAATACGACTCACTATAGGGACGCCGATACAGGACCAGG
07050′-R	CATGCACCGGAGGCCTT
T7-07040′-F	AATTAATACGACTCACTATAGGGGGTTCGCATCGGCATCGAA
07040′-R	CGGGTAGAGAATGGGGTATTCATGA
T7-*motD*′-F	AATTAATACGACTCACTATAGGGGGACCTTGCGTCCTTTCCTGC
*motD*′-R	CATATCCGGATCTCCTGTAGGGA
T7-07035′-F	AATTAATACGACTCACTATAGGGGAAGTCGGCGTAGGAAACCA
07035′-R	CATGACCAGGCTCCCTACTACG
T7-15040′-F	AATTAATACGACTCACTATAGGGGAGGGTGTCAATCCGGAACAT
15040′-R	CATTCCCTTCCTCCTCAGCGT
T7-*cheY*′-F	AATTAATACGACTCACTATAGGGACCCCCAGTATTGGTCGCC
*cheY*′-R	TCTACTTGGTCGAGCCCCAT
16s-F	ACTCCTACGGGAGGCAGCAG
16s-R	ATTACCGCGGCTGCTGG
*csrA*-F	GACATCCGTATCGTGGTGACTC
*csrA*-R	AAGCCAGCGGTCCAGATCC
*flgc*-F	TCCGCCACCAGTTCCGATG
*flgc*-R	CAACGACGCCAGCCACTTG
*pilZ*-F	GGAGTTGCTGAGTGTCTCTGAG
*pilZ*-R	CCATCCGCACGGCAATACC
*motD*-F	CGCAAGCAAGAGGAAGAGTGG
*motD*-R	TGGCATACATGACGACGAAGAAG
*spoR*-F	GTCGCAGGATCGGAAACTCAG
*spoR*-R	GGCAACGCTACAGGTGGTTC

^a^ The homologous sequences used for recombination, the overlapping sequences used for overlapping extension PCR.

## Data Availability

The original contributions presented in this study are included in the article. Further inquiries can be directed to the corresponding authors.
